# Biomarkers of Inflammation, Oxidative Stress, and Endothelial Dysfunction in Early Detection of Diabetic Foot Ulcers

**DOI:** 10.7759/cureus.82174

**Published:** 2025-04-13

**Authors:** S Sangeeta, Chandu Siripuram, Srujana Konka, Krishnakumar Vaithilingam, Panneerselvam Periasamy, Rajesh Kannan Velu, Ramesh Kandimalla

**Affiliations:** 1 Biochemistry, Government Medical College, Karimnagar, Karimnagar, IND; 2 Department of Hospital Medicine, Geisinger Community Medical Center, Scranton, USA; 3 Internal Medicine, Geisinger Wyoming Valley Medical Center, Wilkes-Barre, USA; 4 Microbiology, Bharathidasan University, Tiruchirappalli, IND; 5 Department of Physiology, Government Erode Medical College, Perundurai, IND; 6 Biochemistry, Government Medical College, Narsampet, Warangal, IND

**Keywords:** biomarkers, crp, diabetic foot ulcer, early detection, endothelial dysfunction, inflammation, mmp-9, oxidative stress, south indian population

## Abstract

Background

Diabetic foot ulcers (DFUs) represent a severe complication of diabetes, contributing to increased morbidity, escalating healthcare expenses, and a heightened risk of limb amputation. Early detection is crucial for preventing ulcer progression. Biomarkers offer a non-invasive approach to identifying at-risk individuals. This study evaluates emerging serum biomarkers for early DFU detection in the South Indian population, focusing on inflammation, oxidative stress, and endothelial dysfunction markers.

Materials and methods

A cross-sectional study was carried out at our tertiary care hospital, which is affiliated with Kakatiya Medical College, Telangana, India. The study included 189 diabetic patients, who were classified into three groups: Control (n=63) - individuals with diabetes but no ulcers, Pre-ulcer (n=63) - patients exhibiting pre-ulcerative foot conditions, and ulcer (n=63) - patients with active foot ulcers. Serum biomarkers analyzed included interleukin-6 (IL-6), tumor necrosis factor-alpha (TNF-α), C-reactive protein (CRP) (inflammatory markers), malondialdehyde (MDA) (oxidative stress marker), matrix metalloproteinase (MMP-9) (extracellular matrix degradation), and vascular endothelial growth factor (VEGF), intercellular adhesion molecule (ICAM-1) (endothelial dysfunction markers).

Peripheral neuropathy evaluation was conducted using the Semmes-Weinstein monofilament test (10 g) and vibration perception threshold (VPT) measurement with a biothesiometer. Peripheral arterial disease (PAD) was assessed using the ankle-brachial index (ABI) with a Doppler ultrasound device and classified using Wagner’s grading system. All the analyses were performed using established statistical methods.

Results

The serum concentrations of IL-6, TNF-α, CRP, MDA, and MMP-9 were notably higher in DFU patients (p < 0.05). Among the inflammatory markers, IL-6 (F = 319.75, p < 0.001), TNF-α (F = 186.03, p < 0.001), and CRP (F = 326.12, p < 0.001) showed a progressive increase across groups. MDA (F = 290.95, p < 0.001) reflected elevated oxidative stress, while MMP-9 (F = 356.21, p < 0.001) exhibited the strongest association with DFU severity. In addition, fasting plasma glucose and HbA1c levels were significantly higher in pre-ulcer and ulcer groups compared to controls (p < 0.001), with HbA1c showing strong positive correlations with MMP-9, CRP, and other biomarkers. VEGF and ICAM-1 were markedly elevated in pre-ulcer patients, indicating early vascular impairment. Among all parameters, MMP-9 and CRP demonstrated the highest diagnostic potential for DFU detection.

Conclusion

Findings suggest that systemic inflammation, oxidative stress, and vascular dysfunction play key roles in the pathogenesis of DFUs. Elevated VEGF and ICAM-1 levels in pre-ulcer patients point to early vascular impairment, supporting their potential as early biomarkers. MMP-9 and CRP showed strong correlations with ulcer severity, highlighting their diagnostic utility for early screening. Additionally, HbA1c levels were significantly associated with biomarker elevations, reinforcing the impact of poor glycemic control on DFU progression. However, given the known limitations of HbA1c in anemic individuals, combining it with objective serum biomarkers may enhance diagnostic accuracy and risk stratification. Integrating such a biomarker-based approach into routine diabetes care could enable earlier intervention and reduce DFU-related complications.

## Introduction

Diabetic foot ulcers (DFUs) represent a significant complication of diabetes, leading to elevated morbidity, rising healthcare costs, and a heightened risk of lower limb amputation [1]. The lifetime probability of developing a foot ulcer in diabetic individuals varies between 19% and 34%, with a greater prevalence reported in developing nations, including India [2, 3]. In South India, the rising incidence of diabetes has correspondingly escalated the burden of DFUs [3], underscoring the necessity for early detection and intervention to enhance patient outcomes.

The pathogenesis of DFUs is multifaceted, involving peripheral neuropathy, ischemia, chronic inflammation, oxidative stress, and extracellular matrix degradation [1]. Conventional diagnostic methods mainly rely on clinical evaluations, which often detect ulcers at later stages, reducing the effectiveness of treatment interventions [1-3]. Consequently, there is a critical need for reliable, non-invasive biomarkers capable of identifying individuals at risk for DFUs before the manifestation of overt clinical symptoms.

Recent studies have explored a range of biomarkers linked to DFUs. Inflammatory markers, including interleukin-6 (IL-6), tumor necrosis factor-alpha (TNF-α), and C-reactive protein (CRP), have been studied for their potential in differentiating infected DFUs from non-infected ones [4, 5]. However, their utility in early detection remains inconclusive. Procalcitonin (PCT) has also been evaluated as a potential marker for early infection in DFUs, but findings suggest it offers limited sensitivity in early-stage detection [6, 7].

Oxidative stress markers, such as malondialdehyde (MDA), along with endothelial dysfunction markers like vascular endothelial growth factor (VEGF) and intercellular adhesion molecule-1 (ICAM-1), play a crucial role in the pathophysiology of DFUs [4-7]. These markers reflect underlying processes such as lipid peroxidation and vascular impairment, which are critical in the development and progression of foot ulcers. Matrix metalloproteinase-9 (MMP-9), a zinc-dependent endopeptidase, plays a critical role in the degradation of the extracellular matrix (ECM) during tissue remodeling and wound healing. In the context of DFUs, persistent hyperglycemia, and chronic inflammation have been shown to upregulate MMP-9 expression, leading to excessive ECM breakdown and impaired tissue regeneration. Elevated MMP-9 levels contribute to delayed re-epithelialization, poor granulation tissue formation, and prolonged wound persistence, which are hallmark features of chronic diabetic ulcers. Moreover, several studies have indicated a strong correlation between increased MMP-9 activity and the severity of DFUs, making it a promising biomarker for early detection and monitoring of ulcer progression [4-6]. Despite these associations, there is a paucity of research focusing on the predictive value of these biomarkers in the South Indian diabetic population.

This study seeks to address the existing knowledge gap by evaluating serum levels of key inflammatory, oxidative stress, endothelial dysfunction, and glycemic biomarkers in diabetic patients with and without foot ulcers in South India. By establishing specific biomarker profiles - including HbA1c and fasting plasma glucose - relevant to this population, the research aims to enhance early diagnostic accuracy, support timely clinical interventions, and ultimately reduce the incidence and severity of DFUs.

## Materials and methods

Methodological approach and study setting

This hospital-based cross-sectional study was conducted at Mahatma Gandhi Memorial (MGM) Hospital, Warangal, a tertiary care center affiliated with Kakatiya Medical College, Telangana, India. The study was carried out from November 2021 to December 2023 to evaluate emerging serum biomarkers for early detection of DFUs in a South Indian diabetic population. Given the rising prevalence of diabetes and DFU-related complications in this region, the study aimed to identify non-invasive biomarkers that could facilitate early diagnosis and intervention.

Study population and sample size

A total of 189 patients with T2DM were enrolled using a systematic sampling method. The sample size was calculated using power analysis (effect size = 0.4, α = 0.05, power = 0.8) to ensure statistical validity [8]. Participants were classified into three groups based on clinical assessment and Wagner's ulcer classification system: the control group (n = 63) included diabetic patients without any foot ulcers; the pre-ulcer group (n = 63) comprised patients with pre-ulcerative conditions such as calluses, blisters, or ischemic changes; and the ulcer group (n = 63) included patients with clinically confirmed DFUs. Classification adhered to the International Working Group on the Diabetic Foot (IWGDF) and the American Diabetes Association (ADA) 2020 guidelines [9].

Inclusion criteria

Participants were eligible if they had a confirmed diagnosis of T2DM for at least five years, were between 35 and 75 years of age, and had no previous history of foot ulcers for those categorized into the control and pre-ulcer groups. This five-year threshold was selected based on the likelihood of developing chronic complications such as neuropathy, endothelial dysfunction, and ECM degradation, which contribute to DFU pathogenesis. Control group participants were free from foot ulcers, peripheral neuropathy, and PAD. Ulcer group participants had ulcers classified as Wagner grade 1 or higher [10]. Written informed consent was obtained from all participants prior to study inclusion.

Exclusion criteria

Exclusion criteria were defined in accordance with the National Institute for Health and Care Excellence (NICE) guidelines [11]. Patients were excluded if they had any chronic infections (e.g., tuberculosis, HIV), autoimmune disorders, malignancies, or had undergone recent major surgery. Individuals receiving immunosuppressive therapy, corticosteroids, or chemotherapy were also excluded. To minimize confounding effects on biomarker levels, participants with any acute or recent infections (such as respiratory, urinary tract, or skin infections) within the past four weeks were excluded. Additional exclusions included severe renal impairment (eGFR <30 mL/min/1.73 m²), advanced hepatic disease, recent major cardiovascular events (e.g., myocardial infarction, stroke), and current pregnancy or lactation.

Clinical and laboratory assessments

Participants underwent comprehensive clinical evaluations, including peripheral neuropathy assessment using the Semmes-Weinstein monofilament (10 g) and vibration perception threshold (VPT) via biothesiometer. Peripheral arterial disease (PAD) was screened using the ankle-brachial index (ABI), with a cutoff of <0.9 indicating PAD. Detailed foot examination included ulcer grading, ischemic signs, infection, and callus formation.

Glycemic assessments

Fasting plasma glucose (FPG) and HbA1c levels were measured to evaluate glycemic control and their correlation with biomarker levels and DFU severity. Venous blood samples were collected after an overnight fast of at least 8 hours. Plasma glucose was estimated using the glucose oxidase-peroxidase (GOD-POD) enzymatic method. HbA1c was quantified using high-performance liquid chromatography (HPLC) according to the National Glycohemoglobin Standardization Program (NGSP) standards. These parameters were analyzed for correlation with inflammatory, oxidative stress, and vascular biomarkers.

Biomarker analysis

Under fasting conditions, 5 mL of venous blood was collected from each participant using sterile vacutainers. The samples were allowed to clot undisturbed at room temperature for 30 minutes and subsequently centrifuged at 3000 rpm for 10 minutes to obtain clear serum. The separated serum was immediately aliquoted into sterile, labeled Eppendorf tubes and stored at −80°C until biomarker analysis to preserve sample integrity. The study analyzed key biomarkers associated with inflammation, oxidative stress, endothelial dysfunction, and ECM degradation, including IL-6, TNF-α, CRP, MDA, MMP-9, VEGF, and ICAM-1. Serum CRP levels were measured using high-sensitivity enzyme-linked immunosorbent assay (hs-ELISA). IL-6 and TNF-α were quantified using a multiplex cytokine bead-based immunoassay. MDA levels were assessed using the thiobarbituric acid reactive substances (TBARS) assay, and MMP-9, VEGF, and ICAM-1 were measured using commercially available ELISA kits (R&D Systems, USA), following the manufacturer’s instructions. All assays were performed in duplicate, and strict quality control measures were followed. Standard calibration curves and internal controls were included with each assay run, and intra- and inter-assay coefficients of variation were consistently maintained below 10% to ensure data accuracy and reproducibility.

Statistical analysis

Data analysis was conducted using SPSS version 25.0 (IBM, USA). Normality was assessed using the Kolmogorov-Smirnov test. Results were presented as mean ± SD or median (IQR). Intergroup comparisons were performed using one-way ANOVA with Bonferroni post hoc tests. Receiver operating characteristic (ROC) curve analysis evaluated the diagnostic performance of biomarkers. Pearson’s correlation was used to assess associations between biomarkers and clinical parameters including HbA1c, ABI, and ulcer severity. Laboratory staff were blinded to clinical group assignments during sample processing and analysis to minimize bias.

Ethical considerations

This study adhered to the Declaration of Helsinki (2013) and received ethical clearance from the Institutional Ethics Committee of Kakatiya Medical College, Warangal (Approval No: IEC/KMC/2021/25, dated 21/10/2021). Informed written consent was obtained from all participants. Data confidentiality was maintained per General Data Protection Regulation (GDPR) and Indian Council of Medical Research (ICMR) guidelines.

## Results

Demographic and glycemic status of study participants

The mean age progressively increased across the groups, from 53.9 ± 5.3 years in the control group (n=63) to 58.5 ± 6.9 years in the pre-ulcer group (n=63) and 60.3 ± 7.3 years in the ulcer group (n=63), with the difference being statistically significant (p < 0.001). The duration of diabetes also showed a significant upward trend: 8.2 ± 3.1 years in the control group, 10.2 ± 3.9 years in the pre-ulcer group, and 11.5 ± 5.2 years in the ulcer group (p < 0.001). FPG levels increased significantly from 108.4 ± 14.5 mg/dL (control) to 134.2 ± 18.9 mg/dL (pre-ulcer) and 162.3 ± 22.7 mg/dL (ulcer) (p < 0.001). Similarly, HbA1c levels were elevated in the ulcer group (9.6 ± 1.1%) compared to the pre-ulcer (8.2 ± 0.9%) and control groups (6.9 ± 0.6%) (p < 0.001) (Table 1).

**Table 1:** Demographic and Glycemic Status of Study Participants HbA1c: Glycated hemoglobin

Parameter	Control (n=63)	Pre-ulcer (n=63)	Ulcer (n=63)	p-value
Age (years, Mean ± SD)	53.9 ± 5.3	58.5 ± 6.9	60.3 ± 7.3	<0.001
Duration of Diabetes (years, Mean ± SD)	8.2 ± 3.1	10.2 ± 3.9	11.5 ± 5.2	<0.001
Fasting Plasma Glucose (mg/dL)	108.4 ± 14.5	134.2 ± 18.9	162.3 ± 22.7	<0.001
HbA1c (%)	6.9 ± 0.6	8.2 ± 0.9	9.6 ± 1.1	<0.001
Male (n (%))	36 (57.1%)	41 (65.1%)	32 (50.8%)	0.304
Female (n (%))	27 (42.9%)	22 (34.9%)	31 (49.2%)	0.304
Hypertension (n (%))	32 (50.8%)	33 (52.4%)	51 (81.0%)	0.002

Gender distribution was comparable among groups, with males comprising 36 (57.1%) in the control group, 41 (65.1%) in the pre-ulcer group, and 32 (50.8%) in the ulcer group, while females accounted for 27 (42.9%), 22 (34.9%), and 31 (49.2%), respectively (p = 0.304). The prevalence of hypertension increased significantly with ulcer severity: 32 (50.8%) in the control group, 33 (52.4%) in the pre-ulcer group, and 51 (81.0%) in the ulcer group (p = 0.002) (Table 1).

Serum biomarker levels across study groups

A total of 189 diabetic patients were classified into three groups: control (n = 63), pre-ulcer (n = 63), and ulcer (n = 63). The serum levels of inflammatory, oxidative stress, endothelial dysfunction, and ECM degradation biomarkers were analyzed and compared across these groups. One-way ANOVA identified statistically significant differences in biomarker levels across the three groups (p < 0.001).

Inflammatory biomarkers

Serum levels of IL-6, TNF-α, and CRP showed a progressive increase from the control group to the ulcer group, with statistically significant differences (p < 0.001). IL-6 levels were highest in the ulcer group (9.3 ± 2.5 pg/mL) compared to pre-ulcer (4.8 ± 1.2 pg/mL) and control (2.1 ± 0.5 pg/mL) groups. A similar trend was observed for TNF-α (ulcer: 12.1 ± 3.0 pg/mL, pre-ulcer: 6.9 ± 1.5 pg/mL, control: 3.5 ± 0.7 pg/mL) and CRP (ulcer: 7.8 ± 1.6 mg/L, pre-ulcer: 3.4 ± 0.7 mg/L, control: 1.2 ± 0.3 mg/L). CRP showed the highest diagnostic potential with an area under curve (AUC) of 0.91 in ROC analysis (Table 2).

**Table 2:** Inflammatory and Oxidative Stress Markers IL-6: Interleukin-6; TNF-α: Tumor Necrosis Factor-Alpha; hs-CRP: High-sensitive C-Reactive Protein; MDA: Malondialdehyde; AUC: Area Under Curve; ROC: Receiver Operating Characteristic

Biomarker	Control (Mean ± SD)	Pre-ulcer (Mean ± SD)	Ulcer (Mean ± SD)	F-value	p-value	AUC (ROC Analysis)
IL-6	2.1 ± 0.5	4.8 ± 1.2	9.3 ± 2.5	319.8	<0.001	0.85
TNF-α	3.5 ± 0.7	6.9 ± 1.5	12.1 ± 3.0	186	<0.001	0.83
hs-CRP	1.2 ± 0.3	3.4 ± 0.7	7.8 ± 1.6	326.1	<0.001	0.91
MDA	0.8 ± 0.2	1.9 ± 0.4	4.2 ± 1.1	291	<0.001	0.8

Oxidative stress marker

MDA, a key oxidative stress marker, was significantly elevated in the ulcer group (4.2 ± 1.1 nmol/mL) compared to the pre-ulcer (1.9 ± 0.4 nmol/mL) and control (0.8 ± 0.2 nmol/mL) groups (p < 0.001). ROC analysis indicated moderate diagnostic accuracy (AUC = 0.80).

Extracellular matrix degradation biomarker

MMP-9 exhibited the strongest association with DFU severity, with the highest F-value (F = 356.21, p < 0.001). These levels were significantly higher in the ulcer group (35.7 ± 6.8 ng/mL) compared to the pre-ulcer (22.1 ± 4.3 ng/mL) and control (10.5 ± 2.1 ng/mL) groups (p < 0.001). MMP-9 showed the highest diagnostic accuracy for DFU detection with an AUC of 0.94 (Table 3).

**Table 3:** Extracellular Matrix and Endothelial Dysfunction Biomarkers MMP-9: Matrix Metalloproteinase-9; VEGF: Vascular Endothelial Growth Factor; ICAM-1: Intercellular Adhesion Molecule 1; AUC: Area Under Curve; ROC: Receiver Operating Characteristic

Biomarker	Control (Mean ± SD)	Pre-ulcer (Mean ± SD)	Ulcer (Mean ± SD)	F-value	p-value	AUC (ROC Analysis)
MMP-9	10.5 ± 2.1	22.1 ± 4.3	35.7 ± 6.8	356.21	<0.001	0.94
VEGF	45.3 ± 8.2	68.2 ± 10.5	84.9 ± 12.7	112.54	<0.001	0.78
ICAM-1	120.5 ± 15.4	178.3 ± 20.2	225.6 ± 25.3	134.78	<0.001	0.82

Endothelial dysfunction biomarkers

VEGF and ICAM-1 levels were significantly elevated in the pre-ulcer group compared to the control group, suggesting early vascular impairment. VEGF levels increased from 45.3 ± 8.2 pg/mL (control) to 68.2 ± 10.5 pg/mL (pre-ulcer) and 84.9 ± 12.7 pg/mL (ulcer). ICAM-1 levels were also significantly elevated (control: 120.5 ± 15.4 pg/mL, pre-ulcer: 178.3 ± 20.2 pg/mL, ulcer: 225.6 ± 25.3 pg/mL). Both biomarkers had moderate diagnostic accuracy, with AUC values of 0.78 (VEGF) and 0.82 (ICAM-1).

Receiver operating characteristic (ROC) curve analysis

ROC curves were constructed to evaluate the diagnostic effectiveness of the biomarkers. MMP-9 (AUC = 0.94) and CRP (AUC = 0.91) emerged as the most reliable biomarkers for DFU detection.

Diagnostic accuracy of biomarkers

MMP-9 demonstrates the highest AUC, sensitivity, and specificity, making it the most accurate biomarker (90% overall accuracy). CRP follows closely with an AUC of 0.91 and an overall accuracy of 87%, indicating strong predictive value. IL-6 and TNF-α show moderate performance with AUC values of 0.85 and 0.83, respectively, while MDA, VEGF, and ICAM-1 exhibit relatively lower diagnostic efficacy, with AUCs ranging from 0.78 to 0.82. Among these, VEGF has the lowest accuracy (70%) (Table 4).

**Table 4:** Diagnostic Accuracy of Biomarkers IL-6: Interleukin-6; TNF-α: Tumor Necrosis Factor-Alpha; hs-CRP: High-sensitive C-Reactive Protein; MDA: Malondialdehyde; MMP-9: Matrix Metalloproteinase-9; VEGF: Vascular Endothelial Growth Factor; ICAM-1: Intercellular Adhesion Molecule 1; AUC: Area Under Curve; ROC: Receiver Operating Characteristic; PPV: Positive Predictive Value; Negative Predictive Value

Biomarker	AUC (ROC Analysis)	Sensitivity (%)	Specificity (%)	PPV (%)	NPV (%)	Overall Accuracy (%)
IL-6	0.85	82	79	81	80	81
TNF-α	0.83	80	77	79	78	79
CRP	0.91	88	85	86	87	87
MDA	0.8	76	74	73	75	74
MMP-9	0.94	91	89	90	88	90
VEGF	0.78	72	70	69	71	70
ICAM-1	0.82	78	75	76	77	76

Correlation between biomarkers and clinical parameters

Pearson’s correlation analysis was conducted to examine the association between biomarker levels and clinical parameters, including HbA1c, ABI, and ulcer severity. The results indicated a strong positive correlation between inflammatory biomarkers (IL-6, TNF-α, CRP) and HbA1c and ulcer severity, suggesting that increased systemic inflammation is associated with poor glycemic control and worsening ulcer conditions. IL-6 and TNF-α exhibited a strong positive correlation with HbA1c (r = 0.992 and r = 0.995, respectively), with p-values indicating statistical significance.

**Figure 1:**
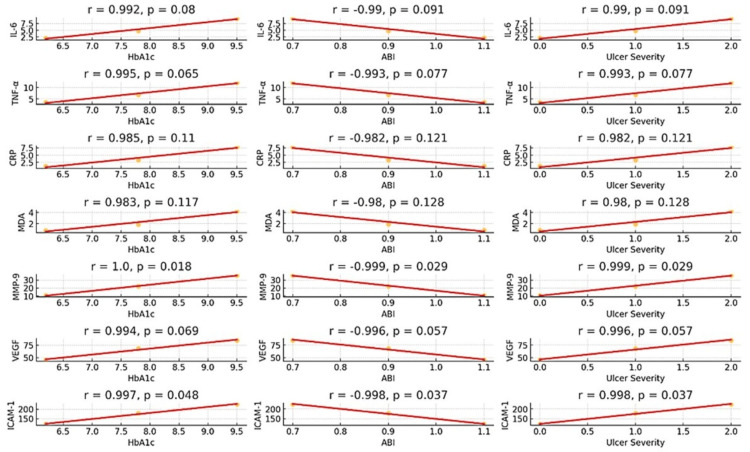
Correlations between biomarkers and clinical parameters (HbA1c, ABI, and Ulcer Severity) IL-6: Interleukin-6; TNF-α: Tumor Necrosis Factor-Alpha; hs-CRP: High-sensitive C-Reactive Protein; MDA: Malondialdehyde; MMP-9: Matrix Metalloproteinase-9; VEGF: Vascular Endothelial Growth Factor; ICAM-1: Intercellular Adhesion Molecule 1

Conversely, a strong negative correlation was found between ABI and inflammatory biomarkers (IL-6, TNF-α, and CRP), suggesting that increased inflammatory responses are associated with peripheral vascular impairment (IL-6 and ABI: r = -0.990; TNF-α and ABI: r = -0.993) (Figure 1). Similarly, oxidative stress markers (MDA) and ECM degradation markers (MMP-9) also showed a positive correlation with ulcer severity, reinforcing their role in wound progression and delayed healing.

## Discussion

DFUs remain a significant complication of diabetes mellitus, contributing to increased morbidity, prolonged hospitalizations, and a high risk of limb amputation [1, 12]. The underlying pathophysiology of DFUs is complex, involving chronic inflammation, oxidative stress, endothelial dysfunction, and ECM degradation [1]. Our study evaluated key serum biomarkers associated with these processes in diabetic patients from a South Indian population. Our findings emphasize the potential diagnostic significance of inflammatory, oxidative stress, and endothelial dysfunction biomarkers in the early detection of DFUs and monitoring disease progression.

Inflammation plays a central role in the pathogenesis of DFUs, contributing to impaired wound healing and increased susceptibility to infection. Our study revealed a significant increase in IL-6, TNF-α, and CRP levels in ulcer patients compared to the pre-ulcer and control groups. These results align with previous research, which highlights IL-6 as a key regulator in the inflammatory cascade, facilitating immune cell activation and cytokine release [13]. Elevated TNF-α levels have also been associated with poor wound healing due to their role in increasing apoptosis and disrupting fibroblast function [14].

Among the inflammatory markers evaluated, CRP demonstrated the highest diagnostic accuracy, underscoring its potential as a reliable screening tool for identifying the progression of DFUs. As an acute-phase reactant produced in response to inflammation and tissue injury, CRP serves as a valuable indicator of ongoing inflammatory processes in diabetic individuals [15]. In the context of diabetes, chronic hyperglycemia initiates a cascade of metabolic and vascular disturbances that promote systemic inflammation. Elevated blood glucose levels generate oxidative stress, which in turn activates pro-inflammatory signaling pathways and upregulates cytokines such as IL-6 and TNF-α. These inflammatory mediators contribute to endothelial dysfunction, leukocyte adhesion, and microvascular injury, all of which impair tissue perfusion and immune regulation. Moreover, prolonged inflammation disrupts the wound-healing cascade by interfering with fibroblast function, collagen synthesis, and angiogenesis [15]. In the present study, a progressive increase in the levels of IL-6, TNF-α, and CRP was observed across the control, pre-ulcer, and ulcer groups, indicating that inflammation plays a critical role in the early pathogenesis of DFUs. These findings highlight the importance of early biomarker-based screening and timely therapeutic interventions to prevent disease progression and improve clinical outcomes.

Oxidative stress plays a central role in the pathogenesis of DFUs by impairing endothelial function, delaying wound healing, and increasing susceptibility to infection [15]. In our study, MDA - a well-established marker of lipid peroxidation - was significantly elevated in DFU patients, indicating heightened oxidative damage during ulcer development. Chronic hyperglycemia in diabetes leads to excessive generation of ROS, which induces peroxidation of polyunsaturated fatty acids in cell membranes, resulting in the formation of MDA. Beyond serving as a marker of oxidative stress, MDA contributes to cellular injury by forming reactive adducts with proteins, nucleic acids, and membrane lipids. This impairs endothelial integrity, disrupts the wound healing cascade, and contributes to tissue necrosis and delayed re-epithelialization. Although MDA exhibited moderate diagnostic accuracy, its progressive increase across study groups supports its relevance in assessing disease severity. Given its association with endothelial dysfunction and impaired angiogenesis - both vital to tissue repair - MDA may serve as a valuable biomarker for monitoring DFU progression. Moreover, these findings highlight the therapeutic potential of antioxidant strategies in improving wound healing outcomes [16, 17].

ECM remodeling is a critical component of the wound healing process, providing the structural support required for cell migration, tissue regeneration, and angiogenesis. Maintaining a balance between ECM synthesis and degradation is essential for orderly progression through the phases of wound repair. MMPs, particularly MMP-9, play a key role in regulating ECM turnover through the controlled breakdown of matrix components. However, in diabetes, this regulatory mechanism becomes disrupted, resulting in excessive ECM degradation. Our study found that MMP-9 levels were significantly elevated in DFU patients (p < 0.001, F = 356.21), with the strongest association observed with ulcer severity. Elevated MMP-9 has been shown to impair re-epithelialization and promote chronic wound persistence by degrading collagen and disrupting tissue architecture [18]. These findings reinforce the role of MMP-9 as both a marker and mediator of delayed wound healing in DFUs.

Notably, MMP-9 demonstrated the highest diagnostic accuracy among the biomarkers analyzed, highlighting its strong potential as an indicator for both the detection and progression monitoring of DFUs. As a critical enzyme involved in ECM remodeling, excessive MMP-9 activity contributes to the degradation of collagen and other structural components of the tissue matrix. This unregulated matrix breakdown compromises tissue integrity, disrupts granulation tissue formation, and significantly impairs the wound healing process [19]. The elevated levels of MMP-9 observed even in the pre-ulcer group further suggest its role in the early stages of ulcer pathogenesis, making it a promising candidate for early risk assessment. Therefore, therapeutic strategies that modulate MMP-9 expression or activity could offer clinical benefit by not only enhancing wound healing but also by potentially preventing the onset or progression of foot ulcers in at-risk diabetic patients.

Endothelial dysfunction is a recognized factor in diabetic complications and plays a crucial role in DFU development. Our study observed significantly elevated levels of VEGF and ICAM-1 in the pre-ulcer group, indicating early vascular impairment preceding ulcer formation. VEGF, a pro-angiogenic factor, was progressively elevated across groups, which is consistent with studies showing that VEGF expression is dysregulated in chronic diabetic wounds [20]. Similarly, ICAM-1, an adhesion molecule involved in endothelial inflammation, was also significantly increased, supporting its role in vascular dysfunction.

The moderate diagnostic accuracy of VEGF suggests their potential as early biomarkers for identifying at-risk individuals before ulcer development. Given that endothelial dysfunction contributes to microvascular complications in diabetes, assessing these biomarkers may help clinicians implement early intervention strategies to prevent DFUs.

The identification of reliable biomarkers for DFU detection holds significant clinical implications. Our findings suggest that a combination of MMP-9, CRP, VEGF, and ICAM-1 may serve as an effective biomarker panel for early risk assessment and ulcer progression monitoring. MMP-9 and CRP demonstrated the highest diagnostic accuracy, making them valuable for distinguishing ulcer patients from at-risk individuals. The elevated levels of MMP-9 and CRP observed in this study can be attributed to chronic hyperglycemia-induced oxidative stress and systemic inflammation commonly seen in diabetes. Hyperglycemia activates pro-inflammatory cytokines such as IL-6 and TNF-α, which in turn stimulate hepatic CRP production and upregulate MMP-9 expression at the site of tissue injury. These processes reflect both systemic inflammatory responses and localized ECM degradation, contributing to delayed wound healing and progression of DFUs. Additionally, VEGF and ICAM-1 may serve as early indicators of vascular impairment, enabling timely intervention to prevent DFU progression.

The strong correlation observed between HbA1c and key biomarkers such as CRP, MMP-9, VEGF, and ICAM-1 underscores the critical role of chronic hyperglycemia in the pathogenesis of DFUs. Persistent elevation in glucose levels leads to oxidative stress and the activation of pro-inflammatory pathways, resulting in systemic inflammation, ECM degradation, and endothelial dysfunction. These pathophysiological changes are reflected in the progressive increase of biomarker levels from control to pre-ulcer and ulcer stages. While detailed foot examination and assessment of neuropathy and PAD remain essential, the integration of HbA1c with these biomarkers provides a more comprehensive risk profile. This approach not only aids in the early identification of high-risk individuals but also reinforces the importance of preventive strategies focused on optimal glycemic control to reduce DFU-related complications.

Incorporating biomarker-based screening into routine diabetes management could facilitate early diagnosis, risk stratification, and personalized therapeutic approaches. This approach has the potential to enhance patient outcomes, lower healthcare costs, and reduce DFU-related complications, including the risk of amputations.

Limitations

Despite its strengths, this study has several limitations. As a cross-sectional study, it does not establish causal relationships between biomarker levels and the progression of DFUs. Future longitudinal studies are needed to evaluate temporal changes in these biomarkers and their predictive value for ulcer development. Additionally, the study was conducted at a single tertiary care center in South India, which may limit the generalizability of the findings to other geographic or demographic populations. While we assessed key biomarkers related to inflammation, oxidative stress, and endothelial dysfunction, other potentially informative markers such as procalcitonin, hypoxia-inducible factors (HIFs), and advanced glycation end products (AGEs) were not included and warrant exploration in future research.

Although glycemic control was evaluated using HbA1c and FPG, it is important to recognize that HbA1c may be unreliable in anemic patients due to altered red blood cell turnover. This limitation highlights the value of integrating objective biomarkers with glycemic parameters for more comprehensive and accurate risk stratification, especially in patients with coexisting anemia. Additionally, the study did not directly compare the diagnostic utility of biomarkers with that of clinical examination or practitioner-based assessment. While standardized physical examination tools such as the Semmes-Weinstein monofilament test and biothesiometry were used to assess peripheral neuropathy, the potential influence of clinician experience on patient evaluation was not analyzed. Incorporating such comparisons in future prospective studies may provide deeper insight into the combined role of clinical judgment and biomarker-based approaches in early DFU detection. Lastly, although the diagnostic utility of the studied biomarkers was established, their potential role in guiding therapeutic interventions - such as anti-inflammatory, antioxidant, or angiogenic therapies - remains unexplored and should be investigated in future clinical trials.

## Conclusions

This study provides compelling evidence that systemic inflammation, oxidative stress, endothelial dysfunction, and ECM degradation play critical roles in the pathogenesis of DFUs. Among the biomarkers assessed, MMP-9 and CRP demonstrated the highest diagnostic accuracy for DFU detection, while VEGF and ICAM-1 showed promise as early indicators of vascular impairment in pre-ulcer patients. Additionally, HbA1c, a widely used marker of long-term glycemic control, exhibited strong positive correlations with inflammatory and oxidative stress markers, further reinforcing the role of chronic hyperglycemia in ulcer progression. The integration of HbA1c with biomarker-based screening may offer a comprehensive approach to early risk stratification and timely intervention.

However, it is important to recognize that HbA1c has certain limitations, particularly in patients with anemia or altered red blood cell turnover, where its values may not accurately reflect glycemic status. In such cases, reliance solely on HbA1c could lead to misclassification of risk. Therefore, combining HbA1c with objective biomarkers such as MMP-9 and CRP can enhance diagnostic accuracy and clinical decision-making.

## References

[1] McDermott K, Fang M, Boulton AJ, Selvin E, Hicks CW (2023). Etiology, epidemiology, and disparities in the burden of diabetic foot ulcers. Diabetes Care.

[2] Das A, Pendsey S, Abhyankar M, Malabade R (2020). Management of diabetic foot in an Indian clinical setup: An opinion survey. Cureus.

[3] Thomas Z, Bhurchandi SK, Saravanan B (2024). Diabetic foot ulcers, their characteristics, and trends in survival: Real world outcomes at a tertiary care facility in India. Diabetes Metab Syndr.

[4] Mohamed AA, Elmotaleb Hussein MA, Nabil Hanna I (2024). The potential impact and diagnostic value of inflammatory markers on diabetic foot progression in type II diabetes mellitus: A case-control study. Med Clin.

[5] Phosat C, Panprathip P, Chumpathat N (2017). Elevated C-reactive protein, interleukin 6, tumor necrosis factor alpha and glycemic load associated with type 2 diabetes mellitus in rural Thais: A cross-sectional study. BMC Endocr Disord.

[6] Omar J, Ahmad NS, Che-Soh N, Wan-Azman WN, Yaacob NM, Abdul-Ghani NS, Abdullah MR (2023). Serum procalcitonin (PCT) - Is there a role as an early biomarker in infected diabetic foot ulcer (IDFU) patients?. Malays Orthop J.

[7] An Y, Xu BT, Wan SR, Ma XM, Long Y, Xu Y, Jiang ZZ (2023). The role of oxidative stress in diabetes mellitus-induced vascular endothelial dysfunction. Cardiovasc Diabetol.

[8] Kadam P, Bhalerao S (2010). Sample size calculation. Int J Ayurveda Res.

[9] Akkus G, Sert M (2022). Diabetic foot ulcers: A devastating complication of diabetes mellitus continues non-stop in spite of new medical treatment modalities. World J Diabetes.

[10] Shah P, Inturi R, Anne D (2022). Wagner's classification as a tool for treating diabetic foot ulcers: Our observations at a suburban teaching hospital. Cureus.

[11] Tan T, Shaw EJ, Siddiqui F, Kandaswamy P, Barry PW, Baker M (2011). Inpatient management of diabetic foot problems: Summary of NICE guidance. BMJ.

[12] Maser RE, Nielsen VK, Bass EB (1989). Measuring diabetic neuropathy. Assessment and comparison of clinical examination and quantitative sensory testing. Diabetes Care.

[13] Aboyans V, Criqui MH, Abraham P (2012). Measurement and interpretation of the ankle-brachial index: A scientific statement from the American Heart Association. Circulation.

[14] Brem H, Tomic-Canic M (2007). Cellular and molecular basis of wound healing in diabetes. J Clin Invest.

[15] Semadi NI (2019). The role of VEGF and TNF-alpha on epithelialization of diabetic foot ulcers after hyperbaric oxygen therapy. Open Access Maced J Med Sci.

[16] Zhang WQ, Tang W, Hu SQ, Fu XL, Wu H, Shen WQ, Chen HL (2022). C-reactive protein and diabetic foot ulcer infections: A meta-analysis. J Tissue Viability.

[17] Vujčić S, Kotur-Stevuljević J, Vekić J (2022). Oxidative stress and inflammatory biomarkers in patients with diabetic foot. Medicina.

[18] Lobmann R, Ambrosch A, Schultz G, Waldmann K, Schiweck S, Lehnert H (2002). Expression of matrix-metalloproteinases and their inhibitors in the wounds of diabetic and non-diabetic patients. Diabetologia.

[19] Li Z, Guo S, Yao F, Zhang Y, Li T (2013). Increased ratio of serum matrix metalloproteinase-9 against TIMP-1 predicts poor wound healing in diabetic foot ulcers. J Diabetes Complications.

[20] Xu J, Gao J, Li H, Zhu Z, Liu J, Gao C (2024). The risk factors in diabetic foot ulcers and predictive value of prognosis of wound tissue vascular endothelium growth factor. Sci Rep.

